# The Obligate Symbiont “*Candidatus* Megaira polyxenophila” Has Variable Effects on the Growth of Different Host Species

**DOI:** 10.3389/fmicb.2020.01425

**Published:** 2020-07-08

**Authors:** Chiara Pasqualetti, Franziska Szokoli, Luca Rindi, Giulio Petroni, Martina Schrallhammer

**Affiliations:** ^1^Dipartimento di Biologia, Università di Pisa, Pisa, Italy; ^2^Mikrobiologie, Institut für Biologie II, Albert-Ludwigs-Universität Freiburg, Freiburg, Germany; ^3^Institut für Hydrobiologie, Technische Universität Dresden, Dresden, Germany; ^4^Dipartimento di Biologia, CoNISMa, Università di Pisa, Pisa, Italy

**Keywords:** brackish, *Paramecium*, stress response, symbiosis, osmolarity, GAMMs

## Abstract

“*Candidatus* Megaira polyxenophila” is a recently described member of *Rickettsiaceae* which comprises exclusively obligate intracellular bacteria. Interestingly, these bacteria can be found in a huge diversity of eukaryotic hosts (protist, green algae, metazoa) living in marine, brackish or freshwater habitats. Screening of amplicon datasets revealed a high frequency of these bacteria especially in freshwater environments, most likely associated to eukaryotic hosts. The relationship of “*Ca*. Megaira polyxenophila” with their hosts and their impact on host fitness have not been studied so far. Even less is known regarding the responses of these intracellular bacteria to potential stressors. In this study, we used two phylogenetically close species of the freshwater ciliate *Paramecium*, *Paramecium primaurelia* and *Paramecium pentaurelia* (Ciliophora, Oligohymenophorea) naturally infected by “*Ca*. Megaira polyxenophila”. In order to analyze the effect of the symbiont on the fitness of these two species, we compared the growth performance of both infected and aposymbiotic paramecia at different salinity levels in the range of freshwater and oligohaline brackish water i.e., at 0, 2, and 4.5 ppt. For the elimination of “*Ca*. Megaira polyxenophila” we established an antibiotic treatment to obtain symbiont-free lines and confirmed its success by fluorescence *in situ* hybridization (FISH). The population and infection dynamics during the growth experiment were observed by cell density counts and FISH. Paramecia fitness was compared applying generalized additive mixed models. Surprisingly, both infected *Paramecium* species showed higher densities under all salinity concentrations. The tested salinity concentrations did not significantly affect the growth of any of the two species directly, but we observed the loss of the endosymbiont after prolonged exposure to higher salinity levels. This experimental data might explain the higher frequency of “*Ca*. M. polyxenophila” in freshwater habitats as observed from amplicon data.

## Introduction

*Paramecium* (Ciliophora, Oligohymenophorea) is a unicellular protist with a broad, nearly global distribution in fresh and brackish water bodies. This ciliate is studied, among other things (Karunanithi et al., 2019; Kelz and Mashour, 2019; Mayne et al., 2019; Soares et al., 2019; Arnaiz et al., 2020) for the abundance and diversity of its endosymbionts (Floriano et al., 2018; Garushyants et al., 2018; Grosser et al., 2018; Potekhin et al., 2018; Sabaneyeva et al., 2018; Schrallhammer et al., 2018; Castelli et al., 2019a, b; Fokin et al., 2019; Koehler et al., 2019; Lanzoni et al., 2019; Plotnikov et al., 2019). Host-symbiont interactions and their outcome have been studied for example using *Holospora* (Lohse et al., 2006; Hori et al., 2008; Fokin and Görtz, 2009; Nidelet et al., 2009; Duncan et al., 2013, 2018; Banerji et al., 2015; Castelli et al., 2015; Dusi et al., 2015; Garushyants et al., 2018; Grosser et al., 2018) *Caedibacter* (Kusch et al., 2002; Dusi et al., 2014; Grosser et al., 2018; Schu and Schrallhammer, 2018; Koehler et al., 2019) and *Preeria* (Bella et al., 2016; Potekhin et al., 2018). Despite an increasing number of studies, our knowledge about the impact of symbionts on *Paramecium* is limited, especially when considering that this ciliate is among the best studied protists in regard to host-symbiont interactions. Less is known regarding the response of these symbiotic systems exposed to additional environmental stressors. Even in a balanced system, the introduction of a stressor can have severe consequences. The impact of a symbiont could either shift towards virulent behavior (Dusi et al., 2014; Bella et al., 2016; Schu and Schrallhammer, 2018) and hence, represents an additional biotic stressor, or alternatively has a positive effect on the host’s stress response and thus on survival, e.g., for salinity stress (Smurov and Fokin, 1998; Duncan et al., 2010) or heat shock (Hori and Fujishima, 2003; Fujishima et al., 2005; Hori et al., 2008; Duncan et al., 2010). Some authors (Fokin and Sabaneyeva, 1990; Smurov and Fokin, 1998) reported a higher bacterial infection frequency in protists, i.e., *Paramecium*, living in brackish environments compared to those living in freshwater environments. This might imply an evolutionary advantage for symbiont-bearing microorganisms in habitats exposed to salinity stress. Members of the genus *Paramecium* are well known to be highly sensitive not only to temperature stress but also to increased salinity concentrations (Duncan et al., 2010). Contrary, it could indicate that stressed protists are more susceptible to infection.

In 2013, a bacterial species of the order *Rickettsiales* has been described as endosymbiont of different ciliates (Schrallhammer et al., 2013). All members of this order are obligate intracellular bacteria hosted by eukaryotic organisms and strictly depend on their hosts for multiplication (Dumler et al., 2001) with the notable exception of the epibiont “*Candidatus* Deianiraea vastatrix” (Castelli et al., 2019a). “*Candidatus* Megaira polyxenophila” is remarkable as it has been found associated to potential hosts spanning a huge diversity, e.g., various ciliates (Sun et al., 2009; Vannini et al., 2005; Schrallhammer et al., 2013; Zaila et al., 2017), amoebae (Hess, 2017) chlorophytes and streptophytes (Hollants et al., 2013; Kawafune et al., 2015; Yang et al., 2016) and even cnidarians (Fraune and Bosch, 2007; Sunagawa et al., 2009). Interestingly, the *Megaira*-infected organisms cover a surprising ecological range from freshwater lakes and ponds to brackish waters and even marine systems. Recently, this bacterium has been found associated with multiple *Paramecium* species, including several members of the *Paramecium aurelia* complex representing a group of phylogenetically very closely related species (Lanzoni et al., 2019). In this work, we found “*Ca*. Megaira polyxenophila” (from here referred as *Ca*. M. polyxenophila) naturally occurring in two species of the *P. aurelia* complex, i.e., *Paramecium pentaurelia* and *Paramecium primaurelia.*

The aim of this study is to analyze the role of *Ca*. M. polyxenophila, determining the symbiont’s effect on the growth of two phylogenetically close species of *Paramecium* after establishment of the corresponding symbiont-free cell lines via antibiotic treatment. The obtained infected and aposymbiotic lines were then used to compare their performance under standard laboratory conditions as well as at different salinity levels corresponding to oligohaline brackish water. Difference in growth performance depending on presence or absence of *Ca*. M. polyxenophila will shed light on the function of this endosymbiont and the role of this symbiosis under different environmental conditions such as increasing osmolality.

## Materials and Methods

### Experimental Organisms and Their Cultivation

Two different *Paramecium* species naturally harboring *Ca*. M. polyxenophila were used as endosymbiont-infected lines and to establish symbiont-free cells. The host species, as indicated in Table 1, were *P. pentaurelia* US_YE9 (from here on referred to as YE9) derived from Bloomington, IN, United States, whereas *P. primaurelia* Rio Lg_Jac 2III (from here on referred to as LgJac), was sampled in Rio de Janeiro, Brazil. Both species are freshwater protists. After antibiotic (AB) treatment (see below), we established aposymbiotic cell lines *viz.* YE9AB and LgJacAB. All cultures were maintained at 19°C in Cerophyll medium (CM) inoculated with *Raoultella planticola* DSM3069 (*Enterobacteriales*, *Enterobacteriaceae*) (Chiellini et al., 2019).

**TABLE 1:** *Paramecium* species used in this study, their endosymbionts and geographic origin.

***Paramecium* species**	**Symbiont**	**Symbionts’ localization**	**Origin**	**Sampled by**
*P. pentaurelia* YE9	*Ca.* M. polyxenophila	Cytoplasm	Bloomington, IN, United States	Yana Eglit
*P. pentaurelia* YE9AB	None		Established in this study	
*P. primaurelia* LgJac	*Ca.* M. polyxenophila	Cytoplasm	Rio de Janeiro, Brazil	Sascha Krenek
*P. primaurelia* LgJacAB	None		Established in this study	

Prior to the experiment, a single cell was isolated from each cell line and was washed several times in spring water from a freshly opened bottle (San Benedetto S. p. A. Italy) in order to minimize the presence of potentially contaminating microorganisms. The cells were then fed daily for 5 days with CM inoculated with *R. planticola* to induce their exponential growth. These monoclonal cultures (both naturally infected and treated) are maintained since more than four years under laboratory conditions.

### Identification of *Paramecium* Species

*Paramecium* species were identified using morphological characteristics (Fokin, 2010) and the cytochrome *c* oxidase 1 gene (COX1, Barth et al., 2006). Total DNA was extracted using a modified Chelex-based protocol as follows: approximately 100 paramecia cells of each culture were washed three times in sterile spring water and transferred in 100 μl spring water into 0.5 ml Eppendorf tubes. The tubes were stored for at least 20 min at −20°C to freeze completely. Later, 100 μl Chelex solution (Bio-Rad Laboratories, Inc., Hercules, CA, United States) was added and the samples were incubated for 20 min at 99°C. Immediately after incubation the tubes were put on ice. PCR products were obtained by using the forward primer M13 (5′-GTA AAA CGA CGG CCA G-3′, Strüder-Kypke and Lynn, 2010) for LgJac and degenerated forward primer F199dT-B (5′-TGT AAA ACG ACG GCC AGT TCA GGW GCT GCM TTA GCH ACY ATG-3′, Strüder-Kypke and Lynn, 2010) for YE9 as well as the degenerated reverse primer R1143dT (5′-CAG GAA ACA GCT ATG ACT ART ATA GGA TGM CCW CCA TAA GC-3′, Strüder-Kypke and Lynn, 2010) for both strains. Purification was performed with the NucleoSpin Gel and PCR Clean-up Kit (Macherey-Nagel GmbH & Co., KG, Düren NRW, Germany), and products were sequenced directly in both directions with the same primers used for amplification at Eurofins Genomics GmbH (Ebersberg, Germany).

In order to confirm their affiliation to the respective *Paramecium* species, the obtained sequences were compared to available *Paramecium* COX1 sequences. A Maximum likelihood (ML) tree was calculated with IQ−TREE (Nguyen et al., 2015) based on 34 sequences from 10 different *Paramecium* species comprising 620 characters. The alignment was trimmed to the length of the shortest sequence. The best-fit evolutionary model (Kalyaanamoorthy et al., 2017) according to Bayesian information criterion (BIC) is TPM2u+F+I+G4. Ultrafast Bootstrap support (BS) with 1000 pseudoreplicates (Hoang et al., 2018) was calculated by IQ-TREE.

### Identification of “*Ca.* Megaira polyxenophila”

Prokaryotic SSU rRNA gene sequences were amplified by a touchdown PCR (Don et al., 1991) applying the following annealing temperatures: 58°C (30 s, 5 cycles), 54°C (30 s, 10 cycles), and 50°C (30 s, 25 cycles). For YE9, the bacterial primer combination Bac16SFor (5′-AAG AGT TTG ATC CTG GCT C-3′; modified from Neilan et al., 1997) and Bac16SRev (5′-TAC GGC TAC CTT GTT ACG AC-3′; Neilan et al., 1997) were used for both, PCR and direct sequencing from both sides. PCR on LgJac was performed with the *Alphaproteobacteria* specific forward primer 16S_F19b 5′-CCT GGC TCA GAA CGA ACG-3′ (Serra et al., 2016) and the Bacteria specific reverse primer 16S_R1522a 5′-GGA GGT GAT CCA GCC GCA-3′ (Serra et al., 2016) and sequenced using the internal primers 16S F343 ND 5′-TAC GGG AGG CAG CAG-3′, 16S R515 ND 5′-ACC GCG GCT GCT GGC AC-3′ and 16S F785 ND 5′-GGA TTA GAT ACC CTG GTA-3′ (Serra et al., 2016) at GATC Biotech AG (Konstanz, Germany).

A comparison with other 16S rRNA gene sequences of *Ca*. M. polyxenophila and *Ca*. Megaira venefica was performed. Therefore, we run a ML analysis with IQ−TREE based on an alignment (ARB program; Ludwig et al., 2004) of 43 sequences comprising 1343 characters, trimmed at both sides to the length of the shortest sequence. The applied best-fit evolutionary model is TIM3+F+G4 (according to BIC) and 1000 pseudoreplicates were performed for Ultrafast BS.

### Fluorescence *in situ* Hybridization

The presence or absence of the endosymbionts was verified by performing fluorescence *in situ* hybridization (FISH) experiments with the universal probe EUB338 (5′-ACT CCT ACG GGA GGC AGC AG-3′) (Amann et al., 1990) in combination with the genus-specific probe Megenus_487 (5′-GCCGGGGCTTTTTCTGTTGGT-3′) detecting *“Ca.* Megaira*”* (Lanzoni et al., 2019). About 20 ciliate cells were collected, washed three times in water and fixed with 2% paraformaldehyde (PFA, final concentration) on slides as described by Szokoli et al. (2016). Hybridization and washing were carried out as described by Lanzoni et al. (2019). Images were obtained with a Leica DMR microscope, equipped with an HBO 50W/AC-L2 fluorescent lamp, a Leica DFC490 video camera, and Leica IM1000 Software (v.1.0).

### Elimination of Endosymbionts via Antibiotic Treatment

The antibiotic treatment was performed in order to obtain genetically identical symbiont-free lines from the infected ones. Symbiont-free cells were obtained from both, *P. pentaurelia* YE9 and *P. primaurelia* LgJac, through antibiotic treatment (YE9AB and LgJacAB, respectively). Therefore, approximately 30 cells of the stock cultures were transferred to 500 μl of tetracycline (Carl Roth, Karlsruhe, Germany) solution (130 μg ml^–1^) and incubated for 24 h at 20°C. Later, the cells were washed four times. Individual cells were incubated again in tetracycline solution at 20°C for 24–48 h and then transferred to CM inoculated with *R. planticola* as food organism. After some rounds of cell division, single cells were treated again with tetracycline for 24 h and subsequently transferred into bacterized CM. The success of the antibiotic treatment was confirmed by FISH.

### Fitness Experiment to Determine the Impact of the Endosymbiont on Host Growth at Different Salinity Conditions

In order to prime the cultures for the fitness experiment at different salinity conditions, *Paramecium* cells were adapted to the different salinity concentrations and fed daily for three days with bacterized CM to induce exponential growth (Supplementary Figure S1).

The cell number for each line was adjusted to approximately the same density (ca. 100 cells ml^–1^) with sterile CM. To set-up this experiment, a mix containing 8 ml of CM (in case of 0 ppt) or 8 ml of salinity stock solution (8 ppt for a final concentration of 2 ppt; 18 ppt for 4.5 ppt), 12 ml bacterized CM and 20 ml *Paramecium* culture (approximately 100 cells ml^–1^) were added, the total volume was set to 40 ml per line. The salinities were chosen to represent two different oligohaline brackish water conditions after a preliminary experiment to test the salinity tolerance of the two freshwater paramecia species (data not shown). This mix was then split into three experimental units for each line per salinity combination with 10 ml each (approximately 50 cells ml^–1^) (Supplementary Figure S1).

Each experimental unit was fed once a week with 3 ml of a 1:4 dilution of the respective salinity stock solution (sterile water, 8 or 18 ppt) and bacterized CM, to sustain the paramecia and to maintain a constant salinity concentration throughout the experiment (either 0, 2, or 4.5 ppt) for a period of 28 days.

**FIGURE 1:**
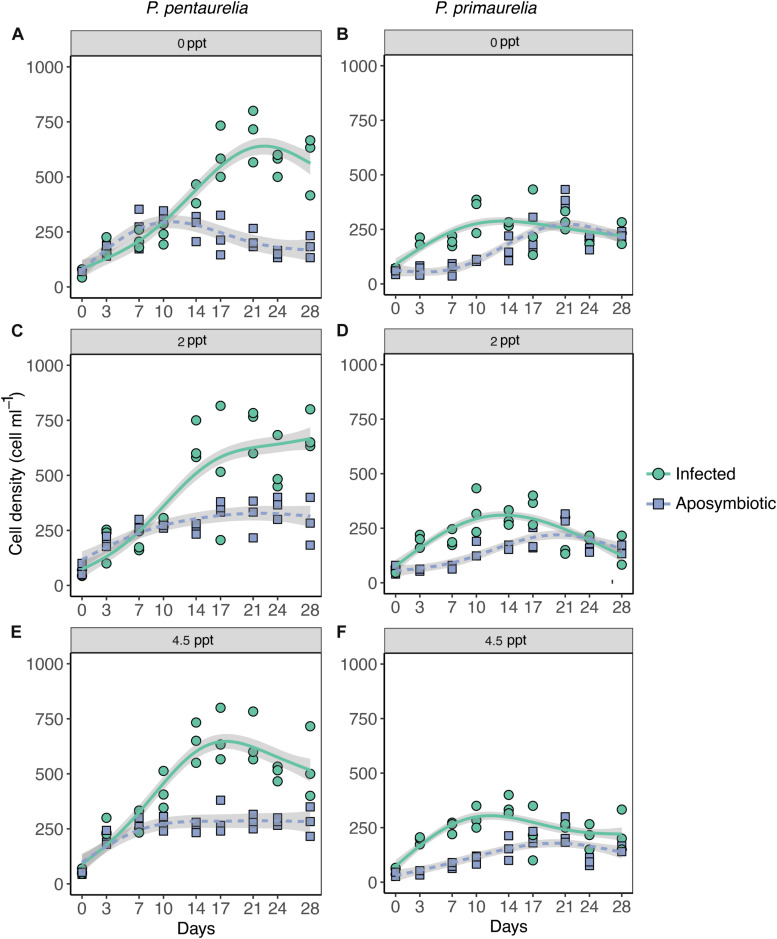
Growth of *Paramecium* lines either infected with *Ca.* Megaira polyxenophila or aposymbiotic at different salinity concentrations over a period of 28 days. *Paramecium pentaurelia* YE9 infected (green line) with *Ca.* Megaira polyxenophila in comparison with the respectively symbiont-free line YE9AB (purple line) at 0 ppt salinity **(A)**, 2 ppt salinity **(C)**, and 4.5 ppt salinity **(E)**. *Paramecium primaurelia* LgJac infected (green line) with *Ca.* M. polyxenophila in comparison with the respectively symbiont-free (purple line) LgJacAB at 0 ppt salinity **(B)**, 2 ppt salinity **(D)**, and 4.5 ppt salinity **(F)**. Data points represent three experimental units and the growth curve is represented by the mean of these three units.

Cell density was determined by counting under a stereomicroscope. The counting days were established as reported in Figure 1 in which T0 is the starting day of the salinity experiment. For each sample, three technical replicates each with a volume of 50–100 μl, depending on the cell number, were counted and the mean of cells per ml was calculated. Samples for cell density estimation were counted and then fixed to perform FISH as described above. These samples were collected twice per week for the duration of the experiment (28 days). Salt stock solutions were prepared by dissolving the corresponding amount of Tropic Marine Salts (Red Sea Salt Meersalz, Red Sea, Düsseldorf, Germany) in deionized water and pasteurizing for 5 min at 95°C.

### Statistical Analysis

The influence of host species with different infectious status and in different salinity settings on *Paramecium* cell density was assessed by using a generalized additive mixed model (GAMM) with gaussian error (R, package: mgcv, version 1.7-28 (Wood, 2017). GAMMs allow assessing the influence of covariates on a response variable without specifying *a priori* fixed-function while accounting for non-independence of data due to successive measures in time. For this reason, time has been included both, in the fixed part, as smoothed term, and in the random part to model dependence. In particular, time has been smoothed independently for different combinations of host species, initial infection status and salinity. Fixed effect terms (infection status, salinity and host species) were modeled using a basis dimension of *k* = 4. The significance of the fixed terms and interactions has been obtained by using the anova.gam function of the mgcv package. The script and dataset are available as Supplementary Table S1 and Supplementary Data Sheet S1. Model accuracy was estimated by the root-mean-square error (RMSE) performing a 10-fold cross-validation on the fixed part of the model using the CVgam function of the gamclass R package. The analysis was performed using R, version 3.6.0.

## Results

### Elimination of *Ca.* M. polyxenophila

**FIGURE 2:**
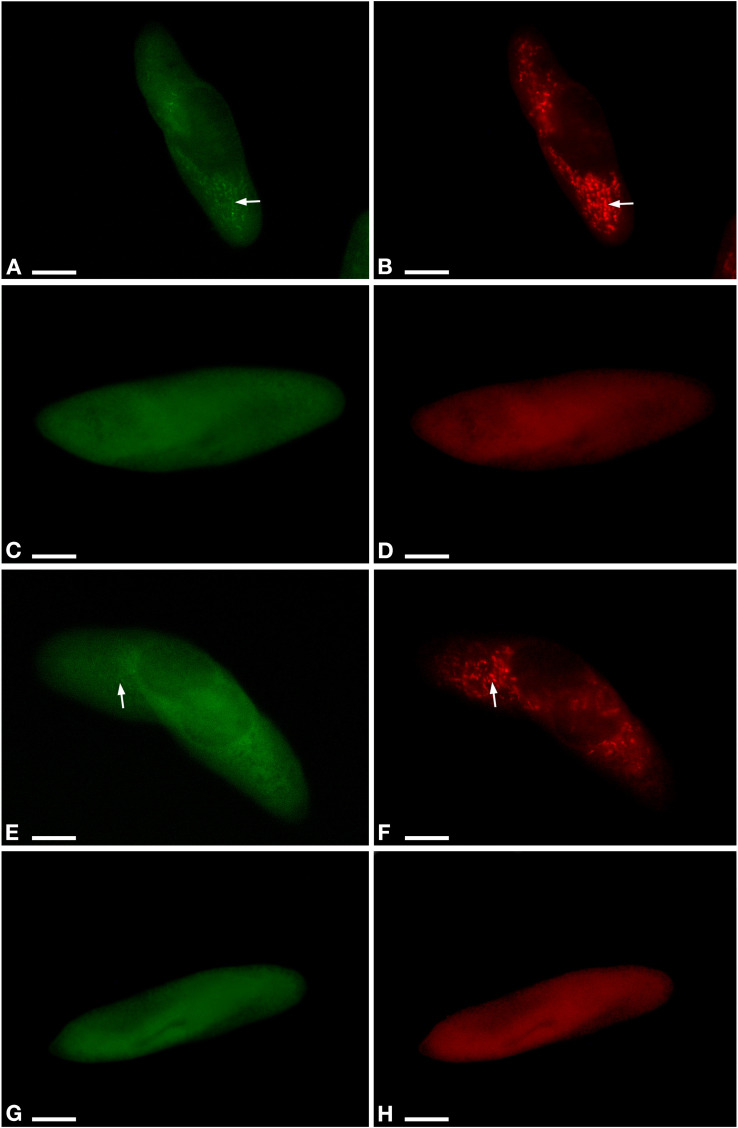
Fluorescent *in situ* hybridization for the visualization of *Ca*. M. polyxenophila in *Paramecium pentaurelia* YE9 **(A,B)** and *Paramecium primaurelia* LgJac **(E,F)**. Fluorescent *in situ* hybridization for the two symbiont-free cell YE9AB **(C,D)** and LgJacAB **(G,H)** after antibiotic treatment using the universal bacterial probe EUB338 **(A,C,E,G)** and Megenus_487 specific for *Ca*. Megaira **(B,D,F,H)**. Arrows indicate *Ca*. M. polyxenophila in the host’s cytoplasm. Scale bar = 10 μm.

Aposymbiotic cells were obtained from both, YE9 and LgJac, through antibiotic treatment producing respectively YE9AB and LgJacAB. A cell was considered successfully treated when no signal with the genus-specific probe Megenus_487 (Lanzoni et al., 2019) was observed from food vacuoles, cytoplasm, or nuclei. Successful elimination of *Ca.* M. polyxenophila was verified repeatedly by FISH (Figure 2). In none of the examined cells, *Ca.* M. polyxenophila was detected after the described tetracycline treatment. Other effects of the antibiotic treatment than symbiont elimination, e.g. delayed or abnormal cell division or population growth, were not observed.

### Confirmation of Host and Endosymbiont Identities

Morphological analyses of the *Paramecium* strains from Bloomington and Rio de Janeiro revealed the strains belonging to the *Paramecium aurelia*-complex. This observation was confirmed by COX1 sequence analysis (Supplementary Figure S2). More precisely, LgJac affiliated with other *P. primaurelia* sequences whereas YE9 belonged to *P. pentaurelia*. Both affiliations received high BS support (98 and 99%, Supplementary Figure S2).

The endosymbiont’s 16S rRNA gene sequences affiliated with other sequences of *Ca*. M. polyxenophila with high BS support (97%, Supplementary Figure S3). The sequence similarity (data not shown) between the type strain of this species (AJ630204) and the here studied intracellular bacteria was 99.93% (LgJac) and 99.55% (YE9).

The obtained sequences are available from NCBI GenBank with following accession numbers: COX1 sequences MT362542 (LgJac) and MT362543 (YE9), 16S rRNA gene sequences MT351038 (LgJac) and MT351039 (YE9).

### Fitness Impact of *Ca.* M. polyxenophila and Salinity on Paramecia

Time included in the model as the smoothed effect revealed a strongly significant effect on estimated degrees of freedom as expected for a nonlinear density growth (Supplementary Data Sheet S1). Among the three fixed factors, host species and infection status showed a highly significant effect (Table 2). Two interaction terms also showed a significant effect, the interaction between infection status and *Paramecium* species and the interaction between salinity and species (Table 2).

**TABLE 2:** ANOVA style-results of the generalized additive mixed models (GAMM) analysis on the salinity, infection, and species on cell density (cell ml^–1^).

	**df**	**F**	***p*-Value**
Salinity	2	1.076	0.343
Infection status	1	27.792	<0.001
Host species	1	49.660	<0.001
Salinity × infection status	2	0.787	0.456
Salinity × host species	2	3.838	0.023
Infection status × host species	1	22.297	<0.001
Salinity × infection status × host species	2	0.333	0.717

*Model accuracy was measured by root-mean-square error (RMSE). *R*^2^ adj. = 0.80; RMES = 79.13.*

According to the GAMM results which revealed a significant effect for host species, a substantial difference in cell densities between the two *Paramecium* species were observed as YE9 grew better in comparison with LgJac, reaching 800 and 400 cell ml^–1^, respectively (Figure 1). At the beginning of the experiment, the starting amount of cells was the same for each line (approximately 50 cells ml^–1^).

The inspection of growth curves and GAMM results indicated that both infected strains showed a generally higher cell density compared to their aposymbiotic counterparts. A strong significant interaction between *Paramecium* species and infection status was caused by the very strong difference in cell density of YE9 (between 600 and 700 cell ml^–1^ at the end of the experiment) compared to YE9AB (200–300 cell ml^–1^) which was not observed in LgJac (Figure 1). The weak significant interaction between salinity and species was due to the density decrease of LgJac and LgJacAB at the higher salinity levels, revealing its stronger salinity stress susceptibility compared to YE9 (Figure 1).

Both *Paramecium* species experienced growth advantages in presence of *Ca.* M. polyxenophila at every salinity condition (Figure 1). There was no obvious general effect of cultivation at oligohaline brackish conditions, neither on infected nor aposymbiotic lines.

### Impact of Salinity on *Ca.* M. polyxenophila Prevalence

**FIGURE 3:**
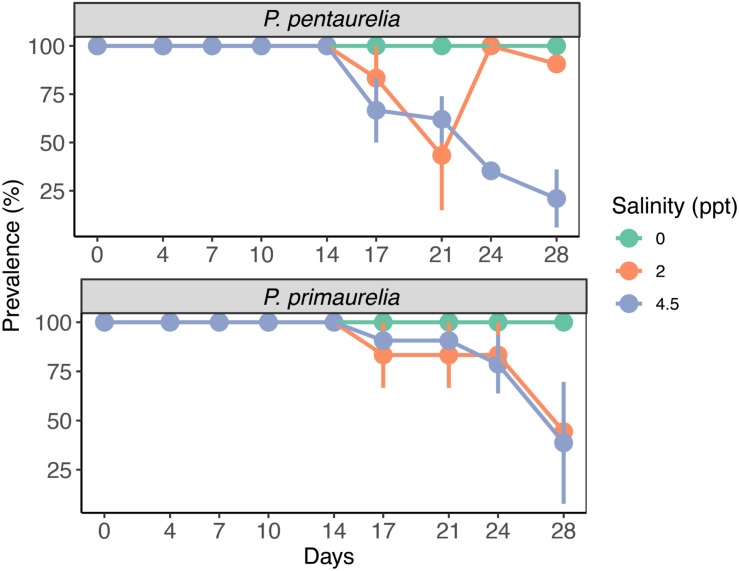
Bacterial prevalence (%) of infected species *P. pentaurelia* and *P. primaurelia* at three different salinity concentrations (0, 2, and 4.5 ppt) over 28 days. Error bars represent the standard error (*n* = 3).

Infected and aposymbiotic paramecia cells of both species were observed by FISH at all sampling time points for all salinity concentrations. The aposymbiotic cells maintained their symbiont-free condition for the complete experiment duration at all tested salinities. FISH results showed that *Ca*. M. polyxenophila was present in YE9 and LgJac at 0 ppt with a prevalence of 100% and in a constant amount at all time points (Figure 3). For the initially *Megaira*-positive cells at 2 and 4.5 ppt, the 100% infection status was maintained until day 17 for both species and declined subsequently. Both species reached the lowest bacterial prevalence at the end of the experiment at 4.5 ppt (Figure 3).

**FIGURE 4:**
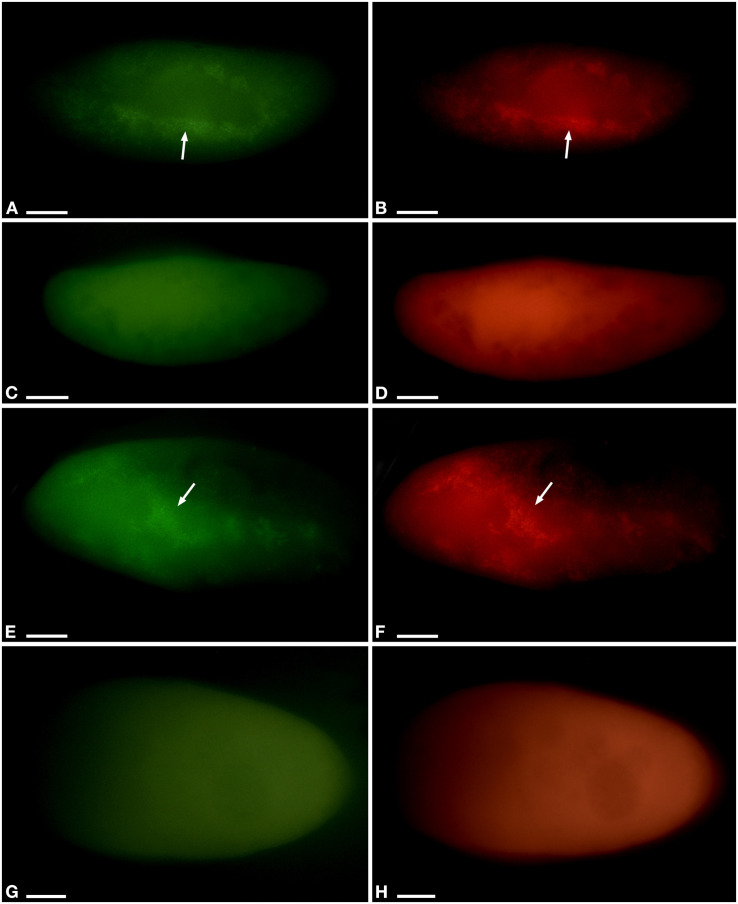
Fluorescent *in situ* hybridization of *Paramecium pentaurelia* YE9 **(A–D)** and *Paramecium primaurelia* LgJac **(E–H)**. Examples for an infected cell **(A,B,E,F)** and one that had spontaneously lost *Ca*. Megaira polyxenophila **(C,D,G,H)** at 4.5 ppt 17 days after the beginning of the growth experiment, using the universal bacterial probe EUB338 specific for 90% of known bacteria **(A,C,E,G)** and Megenus_487 specific for *Ca*. Megaira **(B,D,F,H)**. Arrows indicate *Ca*. M. polyxenophila in the host’s cytoplasm. Scale bar = 10 μm.

While the percentage of *P. pentaurelia* YE9 cells with detected bacterial symbionts continuously declined after day 17 at 4.5 ppt (Figures 3, 4), at 2 ppt the strongest drop in prevalence was observed at day 21. The number of infected cells suddenly recovered and then decreased again. As our approach was to examine approximately 20 cells out of a 1 ml sample at each time point, this strong fluctuation might by chance represent an outlier with such a high number of cells which had spontaneously lost their symbionts.

In case of *P. primaurelia* LgJac, the decrease of *Megaira*-positive cells was very similar at 2 and 4.5 ppt. At the end of the experiment, the infection prevalence dropped to approximately 40% (Figure 3).

## Discussion

Our data suggest that the major effect of *Ca*. M. polyxenophila on *Paramecium* hosts is not harmful. While *Rickettsiaceae* usually are considered as obligate intracellular parasites (DeLong et al., 2014) at least in the case of *Wolbachia* a wide continuum of symbiotic interactions, ranging from mutualistic and essential for host development, fertility, and survival in filarial nematodes to facultative and parasitic in many arthropod species, has been observed (Landmann, 2019). Likely, the fitness effects of *Ca*. M. polyxenophila vary similarly between different hosts and different environmental conditions. Nevertheless, these intracellular bacteria are at least conditionally mutualistic in the two examined *Paramecium* species without providing essential nutritional benefits.

Both *P. pentaurelia* and *P. primaurelia* exhibit improved growth under all tested conditions in presence of the symbiont. Contrary to our expectations, the tested species showed no obvious fitness reduction by increasing salinity levels. This trend indicates that at least the here applied rather mild salt concentrations corresponding to the lower border of oligohaline brackish conditions might be below the physiological stress level of these paramecia. Like many unicellular organisms, *Paramecium* constantly encounter changing environmental conditions e.g., differences in water temperature between seasons, day and night, or even during a day according to the varying solar irradiation. Some fluctuations of environmental conditions remain within the physiological tolerance breadth (Krenek and Berendonk, 2009) whereas others reach stress levels. When paramecia are gradually adapted to stress conditions instead of being suddenly exposed, they can increase their resistance to this particular stressor (Smurov and Fokin, 1998; Tsukuda, 1989). As the cells examined in the fitness assay underwent an acclimatization phase prior to the experiment the applied salinity conditions remained within the organisms’ tolerance range.

Fokin and Sabaneyeva (1990) observed that ciliates living in brackish water apparently harbor more frequently bacterial infections than those in freshwater. We observed a decreasing prevalence of *Ca*. M. polyxenophila starting 2 weeks after the cells were exposed to increased salinity concentrations (2 and 4.5 ppt) while those at freshwater-like conditions remained to 100% infected until the end of the experiment. This is neither a confirmation nor a rejection of the hypothesis that brackish environments favor bacterial infections, but it demonstrates that elevated salt concentrations disrupt the symbiont maintenance in the *Ca*. M. polyxenophila-*Paramecium aurelia* system. Thus, we speculate that long-term exposure to mild environmental stress causes an accumulation of stress signals, for example, an accumulation of misfolded proteins or compatible solutes. While the cellular response of the host will have not necessarily immediate effects on growth, it might either impair bacterial cell division or accidentally cause their expulsion. Alternatively, the increased energy demand in order to adapt to prolonged stress exposure might result in a more directed elimination of the symbionts by lysosomal attack.

As *P. pentaurelia* YE9 and *P. primaurelia* LgJac both benefit from the presence of their symbionts, long-term exposure to brackish salinity conditions will have indirect negative effects on these paramecia.

The loss of *Ca*. M. polyxenophila in brackish conditions might indicate, despite the above mentioned description of this symbiont from marine and brackish host organisms, that they have an adaptation towards freshwater habitats and/or freshwater hosts. This speculation is supported by amplicon sequencing of environmental samples. More than 25% of the amplicon datasets associated with freshwater habitats screened by Lanzoni et al. (2019) revealed positive hits for *Ca*. M. polyxenophila, while the number of marine datasets with signatures of this symbiont was below 5%.

This study provides the first functional analysis of the frequent and promiscuous endosymbiont *Ca*. M. polyxenophila. So far, speculations about a possible opportunistic or parasitic lifestyle of these bacteria (Schrallhammer et al., 2013) are based only on the fact that strains of *Ca*. M. polyxenophila from phylogenetically and geographically distant hosts exhibit very high (99–100%) 16S rRNA gene sequence similarities. This has been considered as strong indication for horizontal transmission capabilities. So far, infection experiments with these bacteria have not been successful under laboratory conditions (Lanzoni et al., 2019). Functional studies regarding the interaction of this symbiont with its host are impaired by (i) its obligate intracellular life style, a trait *Ca*. M. polyxenophila has in common with other members of *Rickettsiales* (Castelli et al., 2016) (ii) the lack of successful protocols for experimental infection experiments, and so far (iii) for symbiont elimination to obtain genetically identical but aposymbiotic (host) organisms. A successful treatment has been established in this study. Similar approaches for generation of the aposymbiotic lines have been used in case of other endosymbionts of *Paramecium* (Kusch et al., 2002; Dusi et al., 2014; Bella et al., 2016; Grosser et al., 2018). The fact that members of *Rickettsiaceae* are naturally resistant to a variety of antibiotics (Rolain et al., 1998) did complicate the establishment of the present protocol. The aposymbiotic lines LgJacAB and YE9AB have been maintained under routine laboratory conditions since at least 5 years. While we cannot completely exclude the small chance that the antibiotic treatment affects *Paramecium* fitness or eliminated free-living bacteria contributing to the observed fitness effects and we would have preferred additional experiments such as infection of naïve cells, those are presently not feasible. Thus, we provide the very first insights into possible effects of *Ca*. M. polyxenophila on its host. As we observed differences in the impact of the endosymbiont in two very closely related host species, it is possible that in more divergent hosts an even broader spectrum of host-symbiont interactions will be observed. As *Ca*. M. polyxenophila infected hosts have been isolated from rather different environments (Lanzoni et al., 2019) the diversity of relevant abiotic factors might further entangle the analysis of the ecological role of this endosymbiont.

## Data Availability Statement

All datasets generated for this study are included in the article/Supplementary Material.

## Author Contributions

CP, FS, and MS designed the research. MS performed the molecular analysis. CP and FS performed the experiments. CP and LR performed the statistical analysis. CP, MS, GP, and LR interpreted the results. CP, FS, MS, and LR wrote the manuscript. All authors critically read and approved the manuscript.

## Conflict of Interest

The authors declare that the research was conducted in the absence of any commercial or financial relationships that could be construed as a potential conflict of interest.

## Supplementary Material

FIGURE S1 Schematic representation of the procedure followed for the fitness experiment.

FIGURE S2 Species affiliation of LgJac and YE9 based on COX1 sequence analysis. Maximum likelihood tree of the genus *Paramecium* calculated with IQ−TREE based on an alignment of 34 sequences from 10 different *Paramecium* species comprising 620 characters. Here obtained sequences are reported in bold. Numbers near nodes indicate Ultrafast Bootstrap support values (below 75% not shown), numbers in brackets indicate sequences in collapsed groups. Scale bar corresponds to 0.1 sequence divergence.

FIGURE S3 Molecular characterization of the endosymbionts of YE9 and LgJac based on 16S rRNA gene sequences. Maximum likelihood tree calculated with IQ−TREE based on 43 sequences belonging to members of the genera “*Ca*. Megaira” and *Rickettsia* comprising 1343 characters. Here obtained sequences are reported in bold. Numbers near nodes indicate Ultrafast Bootstrap support values (below 70% not shown). Scale bar corresponds to 0.01 sequence divergence. *Ca*. stands for *Candidatus*.

TABLE S1 Data sheet for fitness analysis.

DATA SHEET S1 R script.
