# Supplementary figures and images for: The Obligate Symbiont “Candidatus Megaira polyxenophila” Has Variable Effects on the Growth of Different Host Species
Source: Front Microbiol. 2020 Jul 8;11:1425. doi: 10.3389/fmicb.2020.01425 (PMC7360802; doi:10.3389/fmicb.2020.01425)

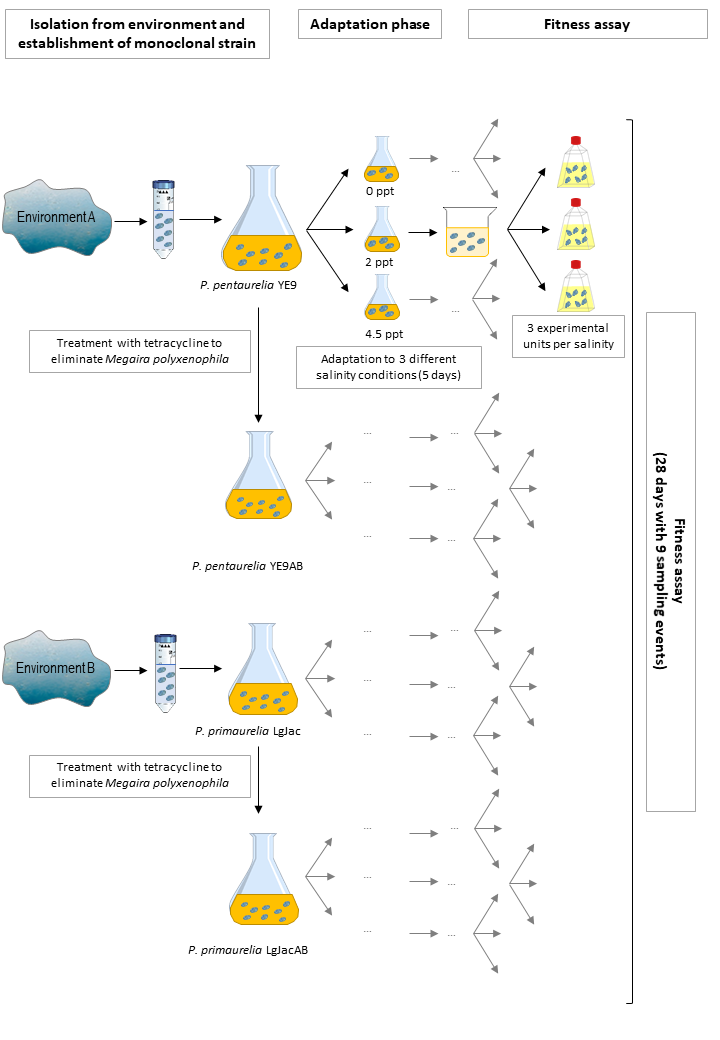

Supplement: FIGURE S1 — Schematic representation of the procedure followed for the fitness experiment. [file Image_1.TIF]

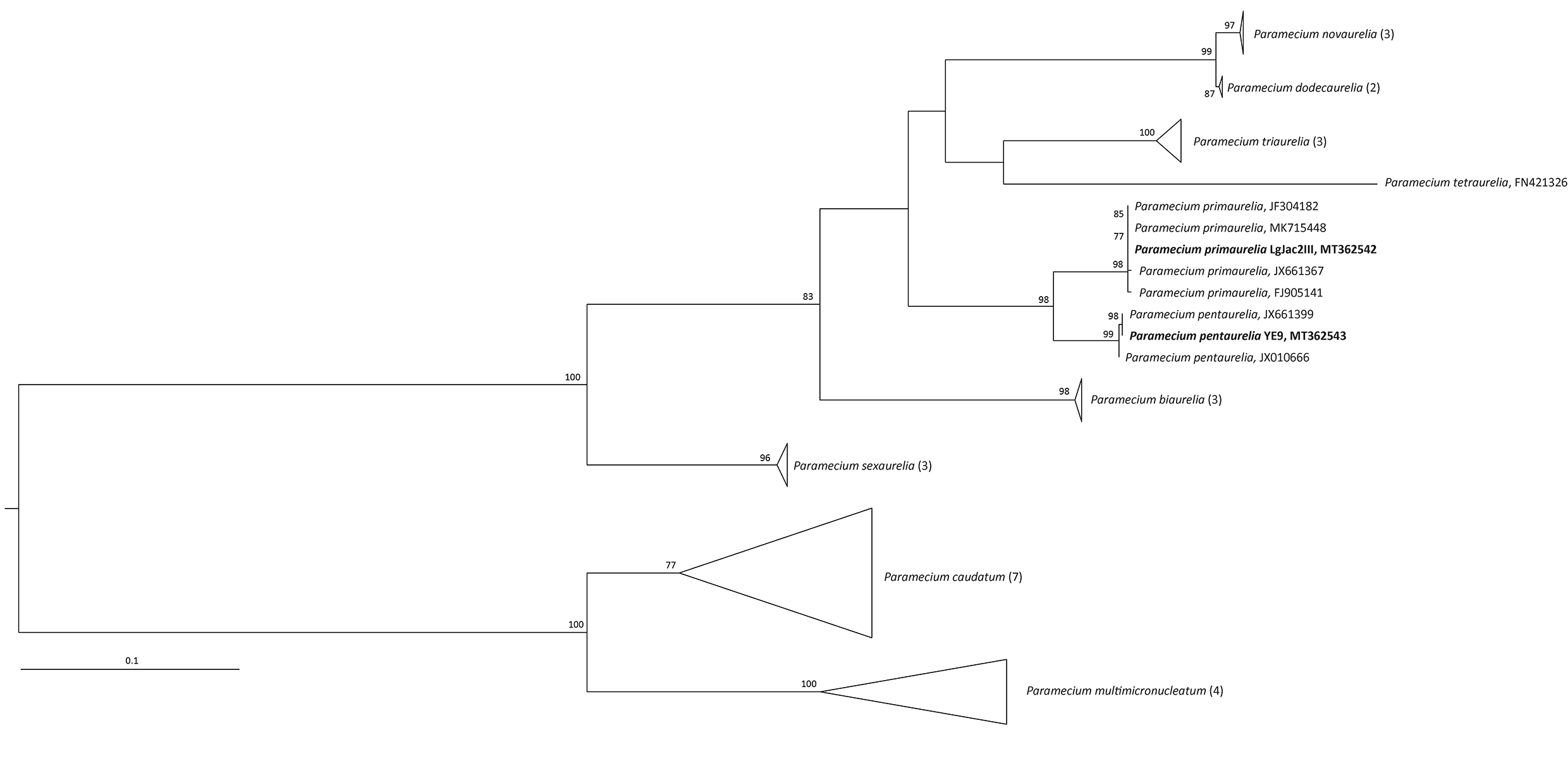

Supplement: FIGURE S2 — Species affiliation of LgJac and YE9 based on COX1 sequence analysis. Maximum likelihood tree of the genus Paramecium calculated with IQ−TREE based on an alignment of 34 sequences from 10 different Paramecium species comprising 620 characters. Here obtained sequences are reported in bold. Numbers near nodes indicate Ultrafast Bootstrap support values (below 75% not shown), numbers in brackets indicate sequences in collapsed groups. Scale bar corresponds to 0.1 sequence divergence. [file Image_2.TIF]

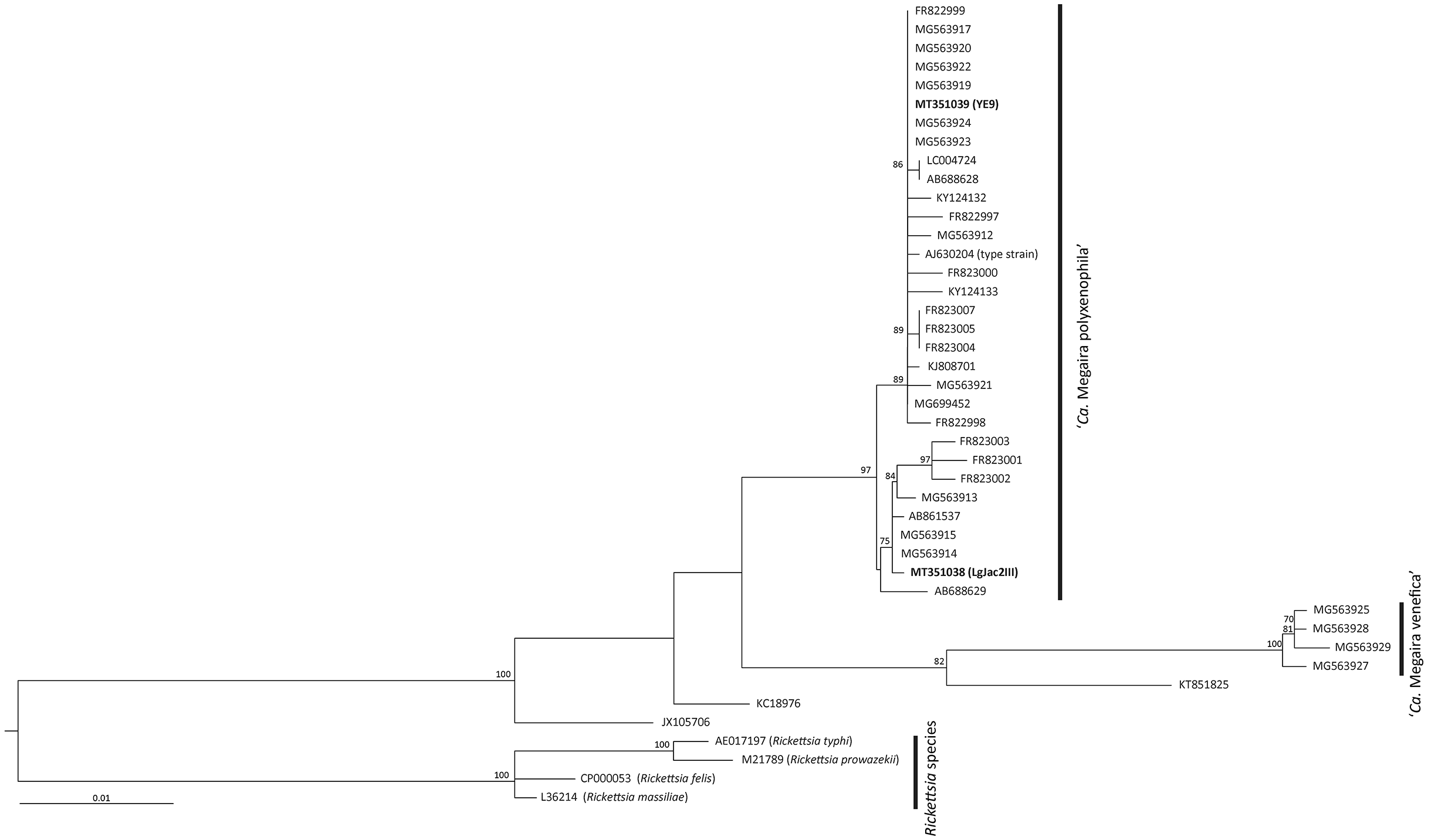

Supplement: FIGURE S3 — Molecular characterization of the endosymbionts of YE9 and LgJac based on 16S rRNA gene sequences. Maximum likelihood tree calculated with IQ−TREE based on 43 sequences belonging to members of the genera “Ca. Megaira” and Rickettsia comprising 1343 characters. Here obtained sequences are reported in bold. Numbers near nodes indicate Ultrafast Bootstrap support values (below 70% not shown). Scale bar corresponds to 0.01 sequence divergence. Ca. stands for Candidatus. [file Image_3.TIF]
