# Supplementary material for: The Obligate Symbiont “Candidatus Megaira polyxenophila” Has Variable Effects on the Growth of Different Host Species
Source: Front Microbiol. 2020 Jul 8;11:1425. doi: 10.3389/fmicb.2020.01425 (PMC7360802; doi:10.3389/fmicb.2020.01425)
Supplement: DATA SHEET S1 — R script. [file Data_Sheet_1.DOCX]

library(mgcv)

library(ggplot2)

# GAM fit

dat.m<-read.table(file.choose(), header = TRUE)

dat.m$salinity<-as.ordered(dat.m$salinity)

dat.m.LgJac<-dat.m[dat.m$species=="LgJac",]

dat.m.LgJac$fitness<-dat.m.LgJac$fitness*10

dat.m.LgJac$salinity<-as.factor(dat.m.LgJac$salinity)

dat.m.LgJac$tr<-as.factor(paste(dat.m.LgJac$AB, dat.m.LgJac$salinity))

p<-ggplot(data=dat.m.LgJac)+

geom_point(data=dat.m.LgJac, aes(x=day, y=fitness

,shape=AB,fill=as.factor(AB)),size=2.7)+

scale_shape_manual(values=c(21,22,23,24))+

scale_fill_manual(values=c("#66C2A5", "#8DA0CB"))+

geom_smooth(data=dat.m.LgJac, aes(x=day, y=fitness, group=Result,linetype=Result,colour=Result),

method="gam",se=T,size=1.5, formula = y ~ s(x, k=4), alpha=0.4)+

scale_colour_manual(values=c("#66C2A5", "#8DA0CB"))+

facet_wrap(~salinity1,scale="free",ncol=1)+

theme_bw()+

theme( panel.grid.minor = element_blank(),panel.grid.major = element_blank(),axis.title.x = element_text(size=12),axis.title.y = element_text(angle=90,size=12),axis.text.x = element_text(size=12),axis.text.y = element_text(face=NULL,size=12))+

theme(panel.border =element_rect(color = 'black'),axis.line = element_line(color = 'black'))+

scale_y_continuous(name=expression("Cell density (cell ml"^-1*")"),limits=c(0,1000))+

scale_x_continuous(name="Days",breaks = unique(dat.m.LgJac$day))

p

dat.m.YE9<-dat.m[dat.m$species=="YE9",]

dat.m.YE9$fitness<-dat.m.YE9$fitness*10

dat.m.YE9$salinity<-as.factor(dat.m.YE9$salinity)

dat.m.YE9$tr<-as.factor(paste(dat.m.YE9$AB, dat.m.YE9$salinity))

p<-ggplot(data=dat.m.YE9)+

geom_point(data=dat.m.YE9, aes(x=day, y=fitness

,shape=AB,fill=as.factor(AB)),size=2.7)+

scale_shape_manual(values=c(21,22,23,24))+

scale_fill_manual(values=c("#66C2A5", "#8DA0CB"))+

geom_smooth(data=dat.m.YE9, aes(x=day, y=fitness, group=Result,linetype=Result,colour=Result),

method="gam",se=T,size=1.5, formula = y ~ s(x, k=4), alpha=0.4)+

scale_colour_manual(values=c("#66C2A5", "#8DA0CB"))+

facet_wrap(~salinity1,scale="free",ncol=1)+

theme_bw()+

theme( panel.grid.minor = element_blank(),panel.grid.major = element_blank(),axis.title.x = element_text(size=12),axis.title.y = element_text(angle=90,size=12),axis.text.x = element_text(size=12),axis.text.y = element_text(face=NULL,size=12))+

theme(panel.border =element_rect(color = 'black'),axis.line = element_line(color = 'black'))+

scale_y_continuous(name=expression("Cell density (cell ml"^-1*")"),limits=c(0,1000))+

scale_x_continuous(name="Days",breaks = unique(dat.m.LgJac$day))

p

##### Prevalence

dat.pre<-read.table(file.choose(), header = TRUE)

dat.pre$species<-unlist(lapply(strsplit(as.character(dat.pre$species),"-"),function(x) x[[1]]))

dat.pre<-dat.pre[,-1]

dat.pre1<-melt(dat.pre, c("species","salinity","rep"))

dat.pre1$day<-as.numeric(gsub("[[:alpha:]]","",as.character(dat.pre1$variable)))

dat.pre1.agg<-aggregate(value~species*salinity*day,data=dat.pre1,mean)

dat.pre1.agg$se<-aggregate(value~species*salinity*day,data=dat.pre1,function(x) std.error(x))$value

dat.m.LgJac$Result<-as.character(dat.m.LgJac$Result)

as.numeric(dat.m.LgJac$Result[!is.na(dat.m.LgJac$Result)])

dat.m.LgJac$Result<-as.numeric()

p<-ggplot(data=dat.pre1.agg)+

geom_point(data=dat.pre1.agg, aes(x=day, y=value

,group=salinity,colour=as.factor(salinity)),size=4)+

geom_line(data=dat.pre1.agg, aes(x=day, y=value, group=salinity,colour=as.factor(salinity)),size=1)+

scale_shape_manual(values=c(21,22))+

scale_fill_manual(values=brewer.pal(5,"Set2"))+

scale_colour_manual(values=brewer.pal(5,"Set2"))+

#geom_path(size=0.5,aes(group=cl,colour=cl))+

geom_errorbar(aes(x=day, ymin=value-se, ymax=value+se,

group=salinity,colour=as.factor(salinity)), width=0, size=1)+

facet_wrap(~as.factor(species),scale="free",ncol=1)+

theme_bw()+

theme( panel.grid.minor = element_blank(),panel.grid.major = element_blank(),axis.title.x = element_text(size=12),axis.title.y = element_text(angle=90,size=12),axis.text.x = element_text(size=12),axis.text.y = element_text(face=NULL,size=12))+

theme(panel.border =element_rect(color = 'black'),axis.line = element_line(color = 'black'))+

scale_y_continuous("Prevalence (%)")+

scale_x_continuous("Days",breaks=unique(dat.pre1.agg$day))

p
